# Family history of mood disorders and concomitant psychopathology in patients with depression

**DOI:** 10.1192/j.eurpsy.2021.907

**Published:** 2021-08-13

**Authors:** E. Kasyanov, A. Nikolishin, G. Rukavishnikov, A. Kibitov, G. Mazo

**Affiliations:** 1 Department Of Translational Psychiatry, V. M. Bekhterev National Medical Research Center for Psychiatry and Neurology, St. Petersburg, Russian Federation; 2 Laboratory Of Molecular Genetics, Serbsky National Medical Research Center on Psychiatry and Addictions, Moscow, Russian Federation

**Keywords:** Depression, anxiety, family history

## Abstract

**Introduction:**

A family history (FH) of mood disorders is an important clinical feature that affects the risk of depression and its clinical manifestations during the course of the disease.

**Objectives:**

To assess the impact of FH in patients with depression on the presence of concomitant psychiatric disorders.

**Methods:**

This cross-sectional study included 172 patients with depression (64.5% women; age - 40,87±15,86 years). The M.I.N.I. was conducted to verify the diagnosis of psychiatric disorders. FH is based on indirect reports of patients.

**Results:**

The most prevalent concomitant psychiatric diagnoses in patients with depression were generalized anxiety disorder (GAD; 26,2%), panic disorder (24,4%) and social anxiety disorder (13,4%). FH was recorded in 52 (30.2%) patients with depression. Patients with depression and FH more often had concomitant GAD (with FH - 20 (38,5%), without FH - 25 (20,8%); p=0.016). Women with depression and FH showed a higher rate of early onset (before age 18) of depression (with FH - 10 (32,3%), without FH - 10 (12,5%); p=0.015). Men with depression and FH more often had concomitant GAD (with FH - 10 (47,6%), without FH - 8 (20%); p=0.025). Logistic regression revealed that FH was associated with GAD in patients with depression (p=0.019).

**Conclusions:**

FH of mood disorders in patients with depression is associated with specific concomitant psychopathology. Further genetic studies are needed to explain this comorbidity.

